# Building an associative classifier with multiple minimum supports

**DOI:** 10.1186/s40064-016-2153-1

**Published:** 2016-04-26

**Authors:** Li-Yu Hu, Ya-Han Hu, Chih-Fong Tsai, Jian-Shian Wang, Min-Wei Huang

**Affiliations:** Department of Psychiatry, Kaohsiung Veterans General Hospital, Kaohsiung, Taiwan, ROC; Department of Information Management, National Chung Cheng University, Chiayi, 62102 Taiwan, ROC; Department of Information Management, National Central University, Jhongli, 32001 Taiwan, ROC; Department of Psychiatry, Chiayi Branch, Taichung Veterans General Hospital, Chiayi, Taiwan, ROC

**Keywords:** Data mining, Classification based on associations, Association rule, Multiple minimum supports

## Abstract

Classification is one of the most important technologies used in data mining. Researchers have recently proposed several classification techniques based on the concept of association rules (also known as CBA-based methods). Experimental evaluations on these studies show that in average the CBA-based approaches can yield higher accuracy than some of conventional classification methods. However, conventional CBA-based methods adopt a single threshold of minimum support for all items, resulting in the rare item problem. In other words, the classification rules will only contain frequent items if minimum support (*minsup*) is set as high or any combinations of items are discovered as frequent if *minsup* is set as low. To solve this problem, this paper proposes a novel CBA-based method called MMSCBA, which considers the concept of multiple minimum supports (MMSs). Based on MMSs, different classification rules appear in the corresponding *minsups*. Several experiments were conducted with six real-world datasets selected from the UCI Machine Learning Repository. The results show that MMSCBA achieves higher accuracy than conventional CBA methods, especially when the dataset contains rare items.

## Background

With the advance of technology in data collection and data processing, enterprises can quickly store large amounts of data. In recent years, data mining has been recognized as a technology that can discover previously unknown and potentially useful information from databases (Witten et al. 2011). Several data mining techniques have been developed, such as association rules mining (Agrawal et al. 1993; Hu and Chen 2006), classification (Cohen 1995; Fernandez-Delgado et al. 2014; Quinlan 1993), clustering (Jain et al. 1999), temporal pattern discovery (Hu et al. 2009; Roddick and Spiliopoulou 2002), and other statistical approaches (Vapnik 1999).

Classification is one of the most important technologies used in data mining. Given a set of data objects as a training set, classification techniques construct classifiers (models) to predict class labels of new data objects. A classifier can be used to infer that a new record belongs to a certain class. Thus far, classification technology has been used in many applications, including customer relationship management, medical diagnosis, and fraud prevention (Jyoti et al. 2011; Ngai et al. 2009; Yoon and Lee 2013). Researchers have developed many classification techniques, which can be categorized as rule-based or non-rule-based approaches. The rule-based approaches, such as decision tree (Quinlan 1993), RIPPER (Cohen 1995), PART (Witten et al. 2011), and classification based on associations (CBA) (Liu et al. 1998, 2000), are typically interpretative and easy to implement. On the other hand, non-rule-based approaches, such as support vector machine (SVM) (Vapnik 1999) and artificial neural network (ANN) (Venkatesh and Thangaraj 2008), have a high noise tolerance but require extensive computation.

Researchers have recently proposed several CBA-based methods, including CMAR (Li et al. 2001), CPAR (Yin and Han 2003), MCAR (Thabtah et al. 2005), CBC (Deng et al. 2014), and MMAC (Thabtah et al. 2004). Experimental studies on these methods show that CBA-based approaches can yield higher accuracy than conventional classification methods. Most CBA-based methods (Li et al. 2001; Thabtah et al. 2004, 2005) adopt a rule selection or pruning techniques to build accurate classifiers by retaining limited but effective rules. These methods typically adopt a single threshold of minimum support for all items (i.e., “item” refers to an *attribute*-*name* associated with a valid *attribute*-*value*), class labels, and itemsets. However, a single minimum support restricts the applicability of current CBA-based methods. Different items of each rule or class label will likely have different levels of importance. For example, some items or class labels may appear frequently in the database, while others may appear rarely. If the minimum support value is set at a high threshold, few items can satisfy this requirement, and rules with rare items cannot be found. To find rules with rare items, the minimum support value must be set relatively low. However, a lower value of minimum support requires extensive computation because the number of combinatorial itemsets increases exponentially; in addition, most of these itemsets are meaningless.

In the past, the class imbalance problem (Guo et al. 2008) has been addressed. In this case, the distribution of class labels is skewed, and thus, the classifiers have poor performance on rare classes. To solve the class imbalance problem, the CBA-based methods (Liu et al. 2000; Janssens et al. 2005) have applied the concept of multiple minimum supports (MMSs) to differentiate class labels. That is, a different *minimum class support* is assigned for each class label. It is worth noting that the above works (Liu et al. 2000; Janssens et al. 2005) only focus on the consideration of MMSs for different class labels. However, to the best of our knowledge, previous studies in classification have not integrated the MMSs into various items. Research in association rule mining has shown that the rare item problem (Liu et al. 1999) produces poor-quality rules. Because the selection of a proper set of classification rules is the primary factor in determining the effectiveness of associative classifiers, it is indispensable to address the rare item problem in CBA-based methods.

In this paper, a new approach for classification with MMSs is proposed to tackle all items and class labels in CBA rule generation. The proposed approach provides a user-defined minimum support for each item and each class label. Because different classification rules appear in the corresponding minimum supports, an algorithm based on the established Multiple Support Apriori (MSapriori) algorithm (Thabtah 2007), called MMSCBA, is proposed to discover a complete set of classification rules with MMSs to build MMSCBA-based classifiers, four methods for classification rule selection are considered. Several experiments were conducted with six real-world datasets from the UCI Machine Learning Repository (http://archive.ics.uci.edu/ml/) to evaluate the performance of these classifiers.

The remainder of this paper is organized as follows. “Related work” section presents related research. “Problem definition” section presents the research problem. “The MMSCBA algorithm” section presents the proposed method. “Experimental evaluation” section presents analysis and discussion. Finally, “Conclusion” section presents the conclusion.

## Related work

### Associative classification

Many studies have shown that associative classification (AC) achieves greater accuracy than other traditional approaches. Several AC-based studies have recently presented classification based on association (CBA) (Liu et al. 1998), classification based on multiple association rules (CMAR) (Li et al. 2001), and classification based on predictive association rules (CPAR) (Yin and Han 2003). An AC-based approach typically consists of three phases: rule generation, rule pruning, and classification.

In the early stage, the CBA approach applies the concept of association rule classification. In CBA, the system initially executes the Apriori algorithm to progressively generate association rules that are satisfied with a user-defined minimum support and confidence threshold. One subset of the generated classification rules becomes the final classifier.

Similarly to CBA, the CMAR approach adopts the FP-Growth algorithm (Guo et al. 2008) to generate frequent itemsets. The subset of matching rules is then used to classify a test object instead of one rule, and this, in turn, improves accuracy. The CMAR approach generates and evaluates rules similarly to CBA; however, CMAR uses a more efficient FP-tree structure. In addition, the CMAR approach considers multiple rules in predicting associated weights. Therefore, CMAR yields higher accuracy than CBA.

Both CBA and CMAR incur a high computation cost in rule generation and rule selection if the dataset is large. To avoid a high computation cost, the CPAR (Yin and Han 2003) approach generates a small set of predictive rules directly from the dataset based on rule prediction and coverage analysis instead of generating candidate rules. The core of CPAR is its predictive rule mining capability, in which an object is correctly covered by a rule instead of being removed. The weight of this object is decreased by multiplying a factor. This is essentially a greedy approach to rule generation and is more efficient than generating all candidate rules. The CPAR approach also uses a dynamic programming approach to avoid repeating calculations during rule generation, allowing it to propose the best *k* rules in prediction. Previous studies have provided more complete surveys of associative classification (Thabtah 2006, 2007; Deen et al. 2010; Swami and Jain 2005).

### Multiple minimum supports

Mining frequent patterns with a single minimum support (abbreviated as *minsup*) implicitly assumes that every item has the same property (i.e., frequency). If the *minsup* value is high, the rules involving rare items will not be found. Conversely, if the *minsup* value is low, a large number of meaningless rules will be generated. The MSapriori (Liu et al. 1999) approach has been proposed to extract frequent rules with rare items. In MSapriori, users are able to discover rare item rules without using frequent items to generate vast numbers of meaningless rules. Based on the definition in (Liu et al. 1999), each item in the database has a *minsup* that is expressed as *minimum item support* (*MIS*), and users can specify different values of *MIS* for different items. This approach makes it possible to observe the nature of the items and their frequencies. The definition of MIS is given as follows.

#### **Definition 1**

Let *I* = {*i*_1_, *i*_2_, …, *i*_*n*_} be a set of items, and let *MIS*(*i*_*p*_) denote the *MIS* value of item *i*_*p*_$(i_{p} \in I)$. The *MIS* value of itemset *A* = {*i*_1_, *i*_2_, …, *i*_*k*_}(1 ≦ *k* ≦ *n*) is defined as follows (Liu et al. 1999).

$$MIS\left( A \right) = min\left[ {MIS\left( {i_{1} } \right),MIS\left( {i_{2} } \right), \ldots ,MIS\left( {i_{k} } \right)} \right]$$

#### *Example 1*

Consider a database including three items: *Milk*, *Granola,* and *Beer*. The user-defined *MIS* values are described as follows:

$$MIS\left( {Milk} \right) = 3\;\% ,MIS\left( {Granola} \right) = 1\;\% ,MIS\left( {Beer} \right) = 0.5\;\%$$

If the support of itemset {*Milk*, *Granola*} is 0.7 %, then itemset {*Milk*, *Granola*} is infrequent because the *MIS* value of itemset {*Milk*, *Granola*} is equal to min[*MIS*(*Milk*), *MIS*(*Granola*)] = 1 %, which is larger than 0.7 %.

In conventional frequent pattern mining, the complete set of frequent patterns satisfies the *downward closure property* if there is only one *minsup*. That is, if an itemset is frequent, then all its subsets are also frequent. However, in the case of MMSs, the *downward closure property* does not hold; that is, certain subsets of a frequent itemset are not frequent and their support values are indeterminate.

#### *Example 2*

Continuing Example 1, the itemset {*Milk*, *Granola*} is infrequent because the support of itemset {*Milk*, *Granola*} is 0.7 %. If the support of itemset {*Milk*, *Granola*, *Beer*} is 0.5 %, then itemset {*Milk*, *Granola*, *Beer*} is frequent because *MIS*(*Beer*) is only 0.5 %. Clearly, the subset of the frequent itemset is not frequent.

To solve this problem, the *sorted closure property* is proposed in (Liu et al. 1999). Suppose that all items in an itemset are sorted in ascending order according to their *MIS* values. The MIS value of any superset of an itemset is equal to that of the first item in this itemset. If an itemset is infrequent based on the MIS value of its first item (i.e., the smallest MIS value among all items in this itemset), then none of its supersets will be frequent. Based on the above property, MSapriori (Liu et al. 1999) can decrease the search space to discover all frequent itemsets with MMSs. Specifically, MSapriori presorts all items according to their *MIS* values but modifies the procedure of generating candidate sets. Because the supports of certain subsets are indeterminate, MSapriori requires post-processing to compute the supports of all subsets of frequent itemsets.

Several extensions of the MSapriori algorithm have been proposed. Hu and Chen (2006) proposed a new data structure, MIS-tree, to enhance the efficiency of MSapriori and to discover frequent patterns with MMSs. The procedure for constructing the MIS-tree only scans a database once. Kiran and Reddy (2010) also proposed an enhanced method. They designed a new method of calculating the *MIS* value called *support difference*. Second, they proposed an FP-growth-like algorithm to extract rare frequent patterns. Finally, they used an evaluation scheme called “item-to-pattern difference” to adjust the distortion if the frequency between each item varies widely. Lee et al. (2005) considered a new perspective on minimum supports. They proposed the concept of maximum constraint, which provides a thorough explanation for certain domains. They also adopted the Apriori-based algorithm to discover large itemsets and association rules within the constraint. Chen et al. (2009) also proposed a fuzzy-based approach called the divide-and-conquer genetic-fuzzy mining algorithm for items with MMSs (DGFMMS). The DGFMMS is designed to find minimum supports, membership functions, and fuzzy association rules.

## Problem definition

Let *I* = {*i*_1_, *i*_2_,…, *i*_*n*_} denote a set of distinct items, where *i*_*p*_ (1 ≤ *p*≤*n*) is an *item* presented in the format of a pair (*attribute*-*name*, *attribute*-*value*). An *event e* is a non-empty set of items, and each item in *e* follows a different attribute-name. Let *Y* be a set of class labels. A rule-item *r* is of the form: *r* = {*e*, *y*}, where *y* is a class label and $y \in Y$.

### **Definition 2**

Given two rule-items $\alpha = \left\{ {(i_{1}^{\alpha } i_{2}^{\alpha } \ldots i_{n}^{\alpha } ),y_{\alpha } } \right\}$ and $\beta = \left\{ {(i_{1}^{\beta } i_{2}^{\beta } \ldots i_{m}^{\beta } ),y_{\beta } } \right\}$ where $y_{\alpha } ,y_{\beta } \in Y$ and $m \le n$ holds. The event in *β*, i.e., $(i_{1}^{\beta } i_{2}^{\beta } \ldots i_{m}^{\beta } )$, is said to be *contained* in *α* if there exist integers 1 ≤ *k*_1_ < *k*_2_ < ··· <*k*_*m*_ ≤ *n* such that $i_{1}^{\beta } = i_{{k_{1} }}^{\alpha }$, $i_{2}^{\beta } = i_{{k_{2} }}^{\alpha }$, …, $i_{m}^{\beta } = i_{{k_{m} }}^{\alpha }$. Moreover, a rule-item *β* is contained in *α* if $(i_{1}^{\beta } i_{2}^{\beta } \ldots i_{m}^{\beta } )$ is contained in *α*, and $y_{\alpha } = y_{\beta }$.

### *Example 3*

Suppose there is a rule-item *α* = {(*a*, 1)(*b*, 2)(*c*, 1)(*b*, 1)(*d*, 2), *y*_1_} and that the rule-item *β* = {(*a*, 1)(*b*, 2)(*c*, 1)(*d*, 2), *y*_1_} is contained in *α* because the relationship between *α* and *β* is satisfied by the two conditions presented previously. As another example, the rule-item *γ* = {(*a,* 1) (*b*, 1) (*d*, 3), *y*_2_} is not contained in *α* because item (*d*, 3) is not included in *α*; that is, condition (1) is not true in the case of *α* and *γ*.

### **Definition 3**

A database *D* consists of a set of records (*id*, *γ*), where *γ* is a rule-item and *id* is the identifier of this rule-item. Given a rule-item $\beta = \left\{ {(i_{1}^{\beta } i_{2}^{\beta } \ldots i_{m}^{\beta } ),y_{\beta } } \right\}$ for rule-item *β* in *D*, define the event support count *e_supp*, the class support count *y_supp* and the rule-item support count *r_supp* as:

$$\begin{aligned} e\_supp_{D} \left( \beta \right)& = |\{ \left( {id,\gamma } \right)|\left( {id,\gamma } \right) \in D \wedge (i_{1}^{\beta } i_{2}^{\beta } \ldots i_{m}^{\beta } )\,{\text{is contained in}}\,\gamma | \hfill \\ y\_supp_{D} \left( \beta \right)& = |\{ \left( {id,\gamma } \right)|\left( {id,\gamma } \right) \in D \wedge y_{\beta } \,{\text{is contained in}}\,\gamma | \hfill \\ r\_supp_{D} \left( \beta \right) &= |\{ \left( {id,\gamma } \right)|\left( {id,\gamma } \right) \in D \wedge \beta \,{\text{is contained in}}\,\gamma | \hfill \\ \end{aligned}$$

### *Example 4*

Table 1 shows all attribute-values for each attribute and the complete set of items. Table 2 shows the sample database *D*. Given a rule-item *β* = {(*a*, 1)(*d*, 2)(*e*, 1), *y*_1_}, the event support count of *β* in *D*, *e_supp*_*D*_(*β*), is 4 (see *sid* 1, 2, 4, and *5*); the class support count of *β* in *D*, *y_supp*_*D*_(*β*), is 3 (see *sid* 1, 4, and *5*); and the rule-item support count of *β* in *D*, *r_supp*_*D*_(*β*), is 3 (see *sid* 1, 4, and 5).

**Table 1:** The attribute-name and attribute-values

Attribute-name	Attribute-value	Item
*a*	{1, 2, 3}	(*a*, 1) (*a*, 2) (*a*, 3)
*b*	{1, 2}	(*b*,1) (*b*, 2)
*c*	{1, 2, 3}	(*c*,1) (*c*, 2) (*c*, 3)
*d*	{1, 2}	(*d*, 1) (*d*, 2)
*e*	{1, 2}	(*e*, 1) (*e*, 2)

**Table 2:** The sample database *D*

*sid*	Database
1	{(*a*, 1)(*b*, 1)(*d*, 2)(*e*, 1), *y*_1_}
2	{(*a*, 1)(*b*, 2)(*c*, 3)(*d*, 2)(*e*, 1), *y*_2_}
3	{(*a*, 3)(*b*, 2)(*c*, 3)(*d*, 1)(*e*, 1), *y*_2_}
4	{(*a*, 1)(*b*, 1)(*c*, 1)(*d*, 2)(*e*, 1), *y*_1_}
5	{(*a*, 1)(*c*, 3)(*d*, 2)(*e*, 1), *y*_1_}

As discussed previously, a single minimum support is inapplicable to real-life cases because of the *rare item problem*. In this paper, the concept of MMSs is introduced, where a user specifies the minimum support threshold of each item.

### **Definition 4**

Let *MIS*(*i*_*p*_) denote the *minimum item support* of item *i*_*p*_, where *i*_*p*_ ϵ *I*. In addition, *MCS*(*y*) represents the *minimum class support* of a class label *y*. Given a rule-item $\beta = \left\{ {(i_{1}^{\beta } i_{2}^{\beta } \ldots i_{m}^{\beta } ),y_{\beta } } \right\}$, the *minimum rule*-*item support* of rule-item *β*, denoted as *MRS*(*β*), is equal to the minimum support value among all items and *MCS*(*y*_*β*_) (i.e., $\hbox{min} (MIS(i_{1}^{\beta } ),MIS(i_{2}^{\beta } ), \ldots ,MIS(i_{m}^{\beta } ),MCS(y_{\beta } ))$).

By using differing minimum item supports for the respective items, users can effectively determine the support requirements for different items. The property of MMSs allows higher minimum supports for the rule-items that only involve frequent items and lower minimum supports for the rule-items that contain rare items.

### **Definition 5**

Given a database *D* and a rule-item $\beta = \left\{ {(i_{1}^{\beta } i_{2}^{\beta } \ldots i_{m}^{\beta } ),y_{\beta } } \right\}$, we call *β* a frequent rule-item if *r_supp*_*D*_(*β*) ≥ *MRS*(*β*). Moreover, the confidence of a frequent rule-item *β* is defined as follows:

$$r\_conf_{D} (\beta ) = \frac{{r\_supp_{D} (\beta )}}{{e\_supp_{D} (\beta )}}.$$

### *Example 5*

Continuing Example 4, the user-specified minimum thresholds are given as follows: *MIS*(*a*, 1) = 3, *MIS*(*a*, 2) = 4, *MIS*(*a*, 3) = 1, *MIS*(*b*, 1) = 3, *MIS*(*b*, 2) = 4, *MIS*(*c*, 1) = 2, *MIS*(*c*, 2) = 1, *MIS*(*c*, 3) = 2, *MIS*(*d*, 1) = 2, *MIS*(*d*, 2) = 3, *MIS*(*e*, 1) = 2, *MIS*(*e*, 2) = 2, *MCS*(*y*_1_) = 2, and *MCS*(*y*_2_) = 1. For a rule-item *β* = {(*a*, 1)(*d*, 2)(*e*, 1), *y*_1_}, the *MRS*(*β*) is equal to min(*MIS*(*a*, 1), *MIS*(*d*, 2), *MIS*(*e*, 1), *MCS*(*y*_1_)) = min(3, 3, 2, 2) = 2. Because *r_supp*_*D*_(*β*) satisfies *MRS*(*β*) (i.e., 3 ≧ 2), we call *β* a frequent rule-item and

$$r\_conf_{D} (\beta ) = \frac{{r\_supp_{D} (\beta )}}{{e\_supp_{D} (\beta )}} = \frac{3}{4} = 0.75.$$

In summary, this approach discovers all frequent rule-items that are satisfied with their own *MRS*. Next, an associative classifier can be built based on the set of all frequent rule-items. For example, a frequent rule-item $\beta = \left\{ {(i_{1}^{\beta } i_{2}^{\beta } \ldots i_{m}^{\beta } ),y_{\beta } } \right\}$ indicates a classification rule $(i_{1}^{\beta } i_{2}^{\beta } \ldots i_{m}^{\beta } ) \to y_{\beta }$ in which the support and confidence are equal to $r\_supp_{D} (\beta )$ and $\frac{{r\_supp_{D} (\beta )}}{{e\_supp_{D} (\beta )}}$, respectively.

## The MMSCBA algorithm

**Fig. 1:**
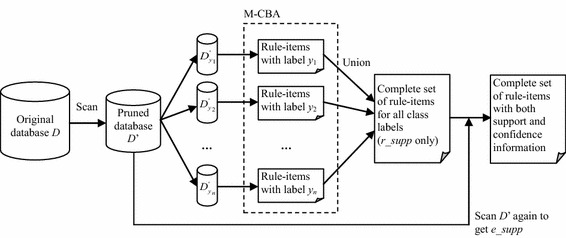
The frequent rule-item generation process

The process of discovering a complete set of frequent rule-items is illustrated in Fig. 1. Initially, scan the complete database *D* once and count the supports of each item. Given the lowest minimum rule-item support *MRS*_*all*_, prune the items not satisfying *MRS*_*all*_ and then form a pruned database *D*’ in which the rule-items are sorted by *MIS* and *MCS* in ascending order. Then, divide *D*’ into partitions, denoted as $D_{y}^{'}$, where each class label *y* satisfies *MCS*. For each partition $D_{y}^{'}$, the *Multiple supports—Classification Based on Associations* (MMSCBA) algorithm is performed to find frequent rule-items. Next, collect all frequent rule-items and their *r_supp*_*D*_ from each partition. Because the database *D’* is divided into separate partitions, scan the entire database to calculate the *e_supp*_*D*_ of the frequent rule-items found in each partition. Finally, all frequent rule-items with their *e_supp*_*D*_ and *r_supp*_*D*_ become classification rules, forming the proposed classifier.

The following subsections depict the MMSCBA algorithm and the scoring approaches for class label prediction.

### The MMSCBA algorithm

**Fig. 2:**
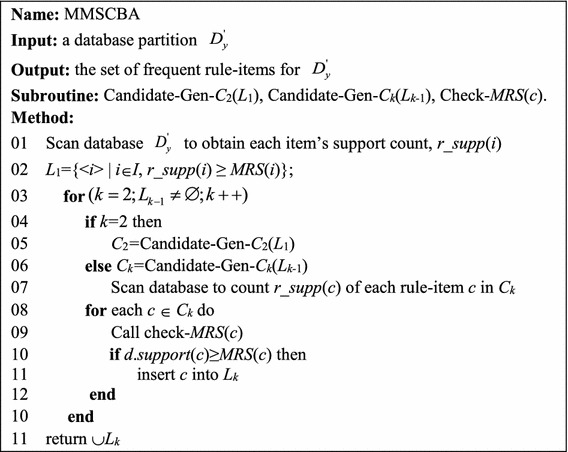
The MMSCBA algorithm

As Fig. 2 shows, the MMSCBA algorithm includes three functions: (1) Candidate-Gen-C_2_(*L*_1_), (2) Candidate-Gen-C_*k*_(*L*_*k*−1_), and (3) Check-*MRS*(*c*).

In Line 1, scan the database $D_{y}^{'}$ to obtain the support count of each item *i*, denoted as *r_supp*(*i*). In Line 2, compare *r_supp*(*i*) with the value of *MRS*(*i*) to determine whether the item *i* is frequent. Each rule-item with an *r_supp*(*i*) value greater than or equal to *MRS*(*i*) is inserted into frequent 1-rule-item set *L*_1_. From Lines 3 to 6, use *L*_*k*−1_ to generate *C*_*k*_. By calling Candidate-Gen-*C*_2_(*L*_1_), use *L*_1_ to generate all 2-candidate-rule-items to form *C*_2_. Similarly, use Candidate-Gen-*C*_*k*_(*L*_*k*−1_) (*k* > 2) to generate all *k*-candidate-rule-items *C*_*k*_ from *L*_*k*−1_. “Candidate-rule-item generation” section details the procedure of candidate rule-item generation. After generating the set of candidate-rule-items, Line 7 scans $D_{y}^{'}$ to obtain the support count of each candidate-rule-item *c*, *r_supp*(*c*). From Lines 8 to 9, use the check-*MRS*(*c*) function to obtain the minimum support of *c*, denoted as *MRS*(*c*). Then, in Lines 10 and 11, the candidate-rule-item *c* with *r_supp*(*c*) ≥ *MRS*(*c*) is inserted into *L*_*k*_. At the end of this stage, we can identify all frequent rule-items from $D_{y}^{'}$.

### Candidate-rule-item generation

From the overview in “The MMSCBA algorithm” section, we can see that the basic concept of the MMSCBA algorithm is similar to the traditional Apriori algorithm (Agrawal et al. 1993). There exists, however, a significant difference between our candidate generation functions and the traditional ones. The main reason for this is that we consider the concept of multiple minimum supports, and the *downward closure property* no longer holds in our approach. In other words, sub-rule-items of a frequent rule-item may not be frequent because the supports of a frequent rule-item and its sub-rule-items may differ. Therefore, to generate a complete set of candidate-rule-items, this study proposes two new candidate generation methods, Candidate-Gen-*C*_2_ and Candidate-Gen-*C*_*k*_, which are based on the definition of MMSs.

**Fig. 3:**
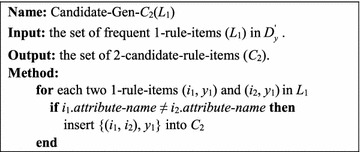
The function Candidate-Gen-*C*_2_(*L*_1_)

Figure 3 presents the function Candidate-Gen-*C*_2_(*L*_1_). Use *L*_1_ to generate *C*_2_ in $D_{y}^{'}$. In *L*_1_, each two frequent 1-rule-items are joined to form a 2-candidate-rule-item. For example, two frequent 1-rule-items (*i*_1_, *y*_1_) and (*i*_2_, *y*_1_) can be joined as a 2-candidate-rule-item, {(*i*_1_, *i*_2_), *y*_1_}. Because all rule-items in $D_{y}^{'}$ have the same class label, we can ignore the class label and only consider the events in two frequent 1-rule-items in the candidate generation process. Note that the attribute-names of *i*_1_ and *i*_2_ cannot be the same (i.e., *i*_1_.*attribute*-*name* ≠ *i*_2_.*attribute*-*name*), and all items in a candidate are sorted in increasing order of their *MIS* values.

**Fig. 4:**
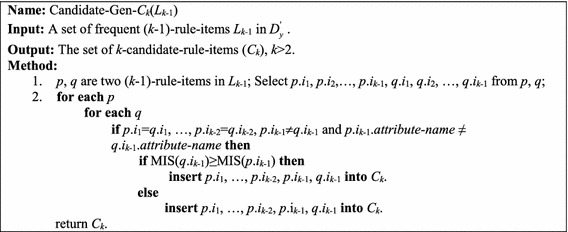
The function Candidate-Gen-*C*_*k*_(*L*_*k*−1_)

**Fig. 5:**
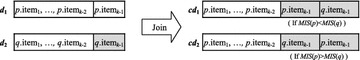
The join method in Candidate-Gen-*C*_*k*_(*L*_*k*−1_)

As Fig. 4 shows, the function Candidate-Gen-*C*_*k*_(*L*_*k*−1_) uses *L*_*k*−1_ to generate *C*_*k*_. Given two (*k−*1)-rule-items *p* and *q*, two *k*-candidate-rule-items (*k* > 2) can be generated if the following two conditions are satisfied: (1) the first (*k* − 2) items of both *p* and *q* are the same; (2) the attribute-names of the last items in *p* and *q* are the same. Figure 5 shows two possible *k*-candidate-rule-items generated by the function Candidate-Gen-*C*_*k*_(*L*_*k*−1_). Note that if the *MIS*(*p.item*_*k*−1_) ≥ *MIS*(*q.item*_*k*−1_) then the *k*-candidate-rule-item *cd*_*1*_ is generated; otherwise, *cd*_*2*_ is generated.

#### *Example 7*

**Fig. 6:**
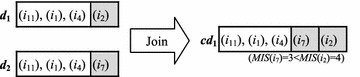
An example of the join method

Continuing Example 4, consider two frequent 4-rule-items with class label *y*_2_, where *d*_1_ = {(*i*_11_)(*i*_1_)(*i*_4_)(*i*_2_), *y*_2_} and *d*_2_ = {(*i*_11_)(*i*_1_)(*i*_4_)(*i*_7_), *y*_2_}. Join the two 4-rule-items to form a new 5-candidate-rule-item in which the first three items in *d*_1_ are identical to those in *d*_2_, but their last items are different. Because *MIS*(*i*_7_) = 3, which is larger than *MIS*(*i*_2_) = 4, a 5-candidate-rule-item *cd*_1_ = {(*i*_11_)(*i*_1_)(*i*_4_)(*i*_7_)(*i*_2_), *y*_2_} can be generated (as shown in Fig. 6).

It is essential that the complete set of frequent patterns can be discovered through the algorithm. Because MMSCBA adopts the candidate-generation-and-test approach to discover all frequent rule-items, the completeness of the candidate generation method needs to be clarified.

Because our approach considers the concept of MMSs, all frequent rule-items must satisfy the sorted closure property. That is, any sub-rule-item *β* of a frequent rule-item *α* is also a frequent rule-item if *MRS*(*β*) = *MRS*(*α*). If *r_supp*(*α*) ≧ *MRS*(*α*), then *r_supp*(*β*) also satisfy *MRS*(*α*) = *MRS*(*β*), i.e., *β* is also a frequent rule-item. This property ensures that our candidate-generation-and-test method is feasible because all possible *k*-candidate-rule-items can be generated from their (*k* − 1)-sub-rule-items.

### Predicting class label based on classification rules

After generating all classification rules, use them to classify uncertain objects in a testing dataset. The prediction of the class labels in associative classification can be categorized into two main approaches: prediction based on the highest precedence single rule-item and prediction based on multiple rule-items. In this study, four prediction measurements are considered: Maximum likelihood (Liu et al. 1998; Thabtah et al. 2005), Max χ^2^ (Li et al. 2001), Laplace (Yin and Han 2003), and Scoring (Hu et al. 2007).

#### Maximum likelihood

Given a testing data object *α* and a set of classification rules, the maximum likelihood approach only considers the highest precedence rule that matches *α*. If there is no applicable rule to match *α*, then the default class label is assigned to *α*. Several associative classification algorithms (Liu et al. 1998; Thabtah et al. 2005) have adopted the maximum likelihood approach for class label prediction.

#### Max χ^2^

Instead of considering a single rule in class label prediction (i.e., Maximum likelihood), the CMAR algorithm (Li et al. 2001) exploits a prediction method that selects a subset of high-confidence rules that are applicable to a class label. The prediction is made by analyzing the correlation among the rules. The correlation is measured using weighted *χ*^2^ analysis to examine the strength of a rule-item based on its support and class frequency in the set of rule-items.

Following Definition 3, the weighted *χ*^2^ of a rule-item, denoted as *Max χ*^2^, is defined as follows:

$$Max\chi^{2} = \left\{ \hbox{min} \left[e\_supp_{D} ,y\_supp_{D} \right] - \frac{{e\_supp_{D} \times y\_supp_{D} }}{|D|} \right\}^{2} \times |D| \times u$$

where

$$u = \frac{1}{{e\_supp_{D} \times y\_supp_{D} }} + \frac{1}{{e\_supp_{D} \times (|D| - y\_supp_{D} )}} + \frac{1}{{(|D| - e\_supp_{D} ) \times y\_supp_{D} }} + \frac{1}{{(|D| - e\_supp_{D} ) \times (|D| - y\_supp_{D} )}}.$$

#### Laplace

Laplace accuracy (Quinlan 1986) is used to estimate the expected accuracy of a rule item. Given a rule-item r, Laplace accuracy can be defined as follows.

$$Laplace\left( r \right) = \frac{{y\_supp_{D} (r) + 1}}{{e\_supp_{D} (r) + |Y|}}$$

where |*Y*| is the number of classes.

To classify a data object, this approach first identifies all matching rule-items and groups them by class labels. For each rule set (i.e., rules having the same class label), the best *k* rules are chosen and then used to calculate the average Laplace accuracy of a class label. Finally, the class label with the highest average Laplace accuracy will be selected as the final prediction outcome.

#### Scoring

Hu et al. (2007) proposes a scoring method to calculate the score of each class label based on all matching rules. Given a frequent rule-item *r*, the two scoring functions are described as follows:

$$\begin{aligned} WeightedSupport(r) & = \frac{{r\_supp_{D} (r )}}{{MRS_{all} }} \\ WeightedConfidence(r) & = r\_conf_{D} (r )\times \frac{{r\_supp_{D} (r)}}{{MRS_{all} }} \\ \end{aligned}$$

The whole procedure of the scoring method can be stated as follows. Given a testing data object *α*, we first identify the complete set of classification rules satisfying *α*, meaning that the event part of a rule-item is a subset of *α*. Next, we divide these rules into sets according to their class labels. The *WeightedSupport* and *WeightedConfidence* of a rule set can be accumulated by summing the score of each rule-item in the set. The class label with the highest *WeightedConfidence* value is selected as the prediction label. If there is more than one class label with the highest *WeightedConfidence* value, then we compare their *WeightedSupport* and choose the class label with the highest value of *WeightedSupport* as the prediction label.

## Experimental evaluation

### Data collection and experimental setup

Six real-world datasets are selected from the UCI machine learning repository website (http://archive.ics.uci.edu/ml/). Table 3 provides a description of these datasets.

**Table 3:** Detailed information of the UCI datasets

Dataset	Attributes	Number of instances	Number of attributes	Number of classes	Rare item problem
Balance Scale (BS)	Discrete	625	4	3	No
Breast Cancer (BC)	Discrete	286	9	2	Yes
Breast Cancer Wisconsin (BCW)	Discrete	699	9	2	Yes
Monks2 (M2)	Discrete	432	6	2	No
Transfusion (TF)	Discrete and continuous	748	4	2	No
Waveform(WF)	Continuous	5000	40	3	No

The experiments were run on a Windows 7 PC equipped with a Intel core i5-4570 3.2 GHz processor and 16 GB of RAM. The proposed methods were implemented using the JAVA language. Several well-known classification techniques were also considered in experimental evaluations, including C4.5, SVM, PART, ANN, RIPPER, and traditional CBA. Among them, C4.5 (Quinlan 1986, 1993), SVM, PART, ANN, and RIPPER were performed using WEKA 3.6.10 (www.cs.waikato.ac.nz/ml/weka) (Witten et al. 2011), a popular suite of machine learning software; the CBA algorithm was performed by adopting its implementation version in (Liu et al. 1998). In all experiments, ten-fold cross-validation (Burman 1989) was adopted to estimate the performance of the proposed method. The accuracy, defined as the proportion of the true results (i.e., both truth positive and truth negative) among the total number of samples examined, was used as the metric to measure the performance of the algorithms.

To easily generate *MIS* values on each item in MMSCBA, we adopted the method proposed in Thabtah (2007), which considers the actual frequencies of items as the basis for MIS value assignment. The equations are stated as follows:

$$\begin{aligned} MIS(i_{p} ) & = \left\{ \begin{array}{ll} M(i_{p} ), & \quad {\text{ if }}M(i_{p} ) \ge MRS_{all} \hfill \\ MRS_{all} , &\quad {\text{ otherwise}} \hfill \\ \end{array} \right. \\ M(i_{p} ) & = \sigma \times f(i_{p} ) \, 0 \le \sigma \le 1 \\ \end{aligned}$$

where *f*(*i*_*p*_) represents the number of times item *i*_*p*_$(i_{p} \in I)$ occurs in the database, and *MRS*_*all*_ denotes the smallest MIS value among all items. *σ* (0 ≦ *σ* ≦ 1) can be used to control the effect of the MIS value in the mining process. In the experiments, we modified the *σ* value from 0 to 1. If *σ* is set to 0, all items will have identical MIS values (i.e., *MRS*_*all*_) and will produce the same results as traditional association rule mining. If *σ* is set to 1 and $M(i_{p} ) \ge MRS_{all}$, $f(i_{p} )$ is the MIS value for *i*_*p*_.

### Results

For every dataset, the value of *minsup* is set as follows: (1) $0.2 \le minsup \le 0.4$ for datasets BS, BC, and BCW; (2) $0.1 \le minsup \le 0.3$ for datasets M2 and TF; and (3) $0.005 \le minsup \le 0.007$ for the dataset WF.

Table 4 presents the classification results of the BS dataset using the MMSCBA with four rule selection methods. The best accuracy of MMSCBA with maximum likelihood, Laplace, scoring, and Max χ^2^ are 0.748, 0.593, 0.708, and 0.384, respectively. MMSCBA with maximum likelihood performs the best compared with the other three classification rule selection methods reported above. MMSCBA with Max χ^2^ has the worst performance.

**Table 4:** The experimental results of dataset BS

minsup	$\sigma$									
	1	0.9	0.8	0.7	0.6	0.5	0.4	0.3	0.2	0.1
Maximum likelihood method										
0.02	0.698	0.701	0.698	0.692	0.681	0.675	0.690	0.705	0.733	0.748
0.03	0.698	0.701	0.698	0.691	0.681	0.678	0.694	0.710	0.742	0.748
0.04	0.711	0.713	0.710	0.703	0.689	0.681	0.695	0.717	0.738	0.739
Laplace method										
0.02	0.434	0.436	0.443	0.445	0.446	0.434	0.432	0.405	0.356	0.296
0.03	0.441	0.443	0.449	0.447	0.449	0.444	0.446	0.413	0.367	0.240
0.04	0.593	0.593	0.587	0.576	0.549	0.562	0.558	0.550	0.538	0.507
Scoring method										
0.02	0.696	0.697	0.699	0.698	0.699	0.691	0.686	0.686	0.678	0.705
0.03	0.691	0.692	0.696	0.697	0.697	0.689	0.680	0.676	0.672	0.708
0.04	0.674	0.674	0.676	0.676	0.675	0.663	0.653	0.646	0.656	0.702
Max χ^2^ method										
0.02	0.269	0.266	0.270	0.271	0.289	0.314	0.327	0.364	0.359	0.369
0.03	0.270	0.267	0.271	0.272	0.290	0.314	0.327	0.364	0.361	0.372
0.04	0.266	0.262	0.267	0.271	0.291	0.323	0.334	0.371	0.380	0.384

For the BC dataset, the results in Table 5 show that the best accuracy of MMSCBA with maximum likelihood, Laplace, scoring, and Max χ^2^ at 0.705, 0.595, 0.706, and 0.624, respectively. MMSCBA with maximum likelihood and scoring methods performs the best compared with the other two methods. MMSCBA with the Laplace method has the worst performance.

**Table 5:** The experimental results of dataset BC

minsup	$\sigma$									
	1	0.9	0.8	0.7	0.6	0.5	0.4	0.3	0.2	0.1
Maximum likelihood method										
0.02	0.644	0.659	0.689	0.693	0.708	0.699	0.707	0.698	0.705	0.704
0.03	0.644	0.659	0.689	0.693	0.707	0.699	0.707	0.698	0.705	0.700
0.04	0.644	0.659	0.689	0.693	0.707	0.699	0.707	0.698	0.704	0.694
Laplace method										
0.02	0.504	0.522	0.573	0.594	0.595	0.569	0.584	0.580	0.543	0.510
0.03	0.504	0.522	0.573	0.594	0.595	0.569	0.584	0.577	0.536	0.513
0.04	0.504	0.522	0.573	0.594	0.595	0.569	0.580	0.575	0.520	0.507
Scoring method										
0.02	0.703	0.703	0.703	0.703	0.703	0.704	0.704	0.703	0.704	0.704
0.03	0.703	0.703	0.703	0.703	0.704	0.704	0.704	0.704	0.703	0.704
0.04	0.703	0.703	0.703	0.703	0.703	0.704	0.704	0.704	0.706	0.706
Max χ^2^ method										
0.02	0.514	0.513	0.558	0.550	0.547	0.590	0.600	0.618	0.624	0.620
0.03	0.514	0.513	0.558	0.550	0.547	0.590	0.600	0.618	0.624	0.613
0.04	0.514	0.513	0.558	0.550	0.547	0.590	0.600	0.618	0.621	0.587

For the BCW dataset, the results in Table 6 show that the best accuracies of MMSCBA with maximum likelihood, Laplace, scoring, and Max χ^2^ were 0.963, 0.950, 0.770, and 0.818, respectively. MMSCBA with the maximum likelihood method performs the best compared with the other three methods. MMSCBA with the scoring method has the worst performance.

**Table 6:** The experimental results of dataset BCW

minsup	$\sigma$									
	1	0.9	0.8	0.7	0.6	0.5	0.4	0.3	0.2	0.1
Maximum likelihood method										
0.02	0.926	0.926	0.923	0.925	0.933	0.945	0.946	0.952	0.953	0.953
0.03	0.948	0.947	0.943	0.942	0.948	0.959	0.959	0.962	0.963	0.963
0.04	0.943	0.942	0.938	0.937	0.944	0.954	0.955	0.959	0.959	0.959
Laplace method										
0.02	0.808	0.823	0.852	0.885	0.916	0.914	0.930	0.944	0.950	0.945
0.03	0.705	0.728	0.795	0.859	0.902	0.906	0.917	0.927	0.923	0.894
0.04	0.776	0.811	0.862	0.895	0.926	0.920	0.922	0.923	0.916	0.912
Scoring method										
0.02	0.752	0.748	0.754	0.763	0.769	0.759	0.766	0.763	0.770	0.767
0.03	0.715	0.713	0.711	0.709	0.708	0.704	0.709	0.709	0.712	0.715
0.04	0.714	0.712	0.715	0.718	0.719	0.720	0.723	0.724	0.726	0.721
Max χ^2^ method										
0.02	0.700	0.688	0.704	0.715	0.737	0.787	0.790	0.795	0.793	0.802
0.03	0.716	0.700	0.715	0.728	0.751	0.804	0.809	0.806	0.814	0.818
0.04	0.787	0.791	0.801	0.805	0.813	0.811	0.804	0.809	0.813	0.818

For the M2 dataset, the results in Table 7 show that the best accuracies of MMSCBA with maximum likelihood, Laplace, scoring, and Max χ^2^ are 0.657, 0.629, 0.672, and 0.604, respectively. MMSCBA with the scoring method performs the best compared with the other three methods. MMSCBA with the Max χ^2^ method has the worst performance.

**Table 7:** The experimental results of dataset M2

minsup	$\sigma$									
	1	0.9	0.8	0.7	0.6	0.5	0.4	0.3	0.2	0.1
Maximum likelihood method										
0.01	0.653	0.657	0.641	0.631	0.616	0.620	0.637	0.640	0.638	0.648
0.02	0.653	0.657	0.641	0.631	0.616	0.620	0.637	0.640	0.638	0.648
0.03	0.653	0.657	0.641	0.631	0.616	0.620	0.637	0.640	0.637	0.645
Laplace method										
0.01	0.626	0.623	0.629	0.625	0.636	0.617	0.610	0.628	0.586	0.460
0.02	0.626	0.623	0.629	0.625	0.636	0.617	0.610	0.628	0.586	0.459
0.03	0.626	0.623	0.629	0.625	0.636	0.617	0.610	0.628	0.586	0.450
Scoring method										
0.01	0.671	0.671	0.671	0.671	0.671	0.671	0.671	0.671	0.672	0.671
0.02	0.671	0.671	0.671	0.671	0.671	0.671	0.671	0.671	0.672	0.671
0.03	0.671	0.671	0.671	0.671	0.671	0.671	0.671	0.671	0.672	0.672
Max χ^2^ method										
0.01	0.575	0.579	0.513	0.516	0.503	0.509	0.551	0.556	0.595	0.603
0.02	0.575	0.579	0.513	0.516	0.503	0.509	0.551	0.556	0.595	0.604
0.03	0.575	0.579	0.513	0.516	0.503	0.509	0.551	0.556	0.596	0.597

For the TF dataset, the results in Table 8 show that the best accuracies of MMSCBA with maximum likelihood, Laplace, scoring, and Max χ^2^ are 0.759, 0.571, 0.762, and 0.730, respectively. MMSCBA with the scoring method performs the best compared with the other three methods. MMSCBA with the Laplace method has the worst performance.

**Table 8:** The experimental results of dataset TF

minsup	$\sigma$									
	1	0.9	0.8	0.7	0.6	0.5	0.4	0.3	0.2	0.1
Maximum likelihood method										
0.02	0.759	0.759	0.759	0.759	0.757	0.758	0.758	0.758	0.758	0.759
0.03	0.759	0.759	0.759	0.759	0.757	0.758	0.758	0.758	0.758	0.758
0.04	0.759	0.759	0.759	0.759	0.757	0.758	0.758	0.758	0.758	0.758
Laplace method										
0.02	0.571	0.549	0.526	0.511	0.523	0.538	0.548	0.494	0.504	0.472
0.03	0.571	0.549	0.526	0.511	0.523	0.538	0.548	0.494	0.504	0.519
0.04	0.571	0.549	0.526	0.511	0.523	0.538	0.548	0.494	0.504	0.516
Scoring method										
0.02	0.762	0.762	0.762	0.762	0.762	0.762	0.762	0.762	0.762	0.762
0.03	0.762	0.762	0.762	0.762	0.762	0.762	0.762	0.762	0.762	0.762
0.04	0.762	0.762	0.762	0.762	0.762	0.762	0.762	0.762	0.762	0.762
Max χ^2^ method										
0.02	0.257	0.342	0.640	0.679	0.717	0.722	0.724	0.727	0.726	0.730
0.03	0.257	0.342	0.640	0.679	0.717	0.722	0.724	0.727	0.726	0.726
0.04	0.257	0.342	0.640	0.679	0.717	0.722	0.724	0.727	0.726	0.726

In summary, the above results of the first five datasets show that MMSCBA with the maximum likelihood method has the highest accuracy, and MMSCBA with the Max χ^2^ method has the lowest accuracy on average. The accuracy of MMSCBA with the scoring method is relatively stable for various values of *α* and *minsup*. The MMSCBA with the Max *χ*^2^ method achieves better accuracy as the value of *α* decreases, but its performance is sensitive to *α*.

Table 9 presents the results of a comparison between non-rule-based classifiers (i.e., ANN and SVM) and rule-based classifiers (i.e., C4.5, PART, RIPPER, CBA, and MMSCBA with maximum likelihood). The results show that the performance of the rule-based classifiers is stable but not the best among all techniques. The accuracy of the non-rule-based classifiers is higher than that of the rule-based classifiers in most of the six datasets.

**Table 9:** Classification accuracy (%) for all classification techniques

Data set	Non-rule-based		Rule-based								
	ANN	SVM	C4.5	PART	RIPPER	CBA (minsup)			MMSCBA (minsup)		
BS	97.92	90.24	64.48	77.28	71.84	71.88 (0.02)	71.40 (0.03)	73.32 (0.04)	74.78 (0.02)	74.85 (0.03)	73.92 (0.04)
BC	67.48	68.18	74.83	72.38	71.68	63.29 (0.02)	59.44 (0.03)	63.29 (0.04)	70.40 (0.02)	70.00 (0.03)	69.42 (0.04)
BCW	95.85	95.85	94.42	93.85	94.13	95.71 (0.02)	95.56 (0.03)	94.84 (0.04)	95.32 (0.02)	96.34 (0.03)	95.93 (0.04)
M2	71.99	44.21	47.92	56.02	51.16	56.06 (0.01)	57.67 (0.02)	55.84 (0.03)	64.79 (0.01)	64.76 (0.02)	64.45 (0.03)
TF	76.34	76.20	76.20	76.34	76.20	76.42 (0.01)	76.42 (0.02)	76.42 (0.03)	75.94 (0.01)	75.85 (0.02)	75.85 (0.03)
WF	82.78	85.96	76.5	78.08	75.94	79.98 (0.005)	79.16 (0.006)	77.41 (0.007)	71.07 (0.005)	78.99 (0.006)	83.79 (0.007)

Table 10 presents the results of runtime for all classifiers and datasets. The results show that the CBA and our approach require more runtime than other classification techniques such as SVM, C4.5, PART, and RIPPER. The results are as expected. It is because the runtime of association rule-based approaches (i.e., CBA and MMSCBA) is affected by the *minsup*. That is, it may require longer execution time when *minsup* is set too low. Therefore, compared to other heuristic approaches (e.g., C4.5, PART, and RIPPER etc.), our approach requires more execution time for discovering all possible classification rules from the datasets.

**Table 10:** Runtime (s) for all classification techniques

Dataset	Non-rule-based		Rule-based				
	ANN	SVM	C4.5	PART	RIPPER	CBA (minsup)	MMSCBA (minsup)
BS	1.30	0.04	0.01	0.01	0.02	0.42 (0.4)	1.24 (0.4)
BC	1.14	0.03	0.01	0.01	0.01	5.35 (0.4)	7.40 (0.4)
BCW	24.22	0.15	0.02	0.01	0.11	6.78 (0.4)	21.42 (0.4)
M2	0.53	0.01	0.01	0.01	0.01	0.36 (0.3)	1.18 (0.3)
TF	0.27	0.01	0.01	0.01	0.01	0.47 (0.3)	1.87 (0.3)
WF	189.09	7.39	0.03	0.28	0.55	20.90 (0.007)	195.68 (0.007)

In many real-life applications, non-rule-based classification techniques cannot be adopted due to low interpretability. In contrast, rule-based classification techniques can generate IF–THEN rules, which can be easily stored in a knowledge base. The expert systems can also be easily built by incorporating the rules into an expert system shell. Therefore, while the performance of rule-based classifiers is acceptable, most decision makers would select rule-based classifiers in practice.

Among all the rule-based classifiers, the experimental results also show that the proposed method (i.e., MMSCBA with the maximum likelihood method) outperforms the traditional CBA and other rule-based techniques in three of the six datasets. Compared with other classification methods, the proposed method achieves remarkable accuracy when the dataset contains rare items, such as the BC and BCW datasets. Although C4.5 and CBA perform the best in datasets BC and TF, respectively, MMSCBA with the maximum likelihood method still has a satisfactory performance (i.e., close to the best classifier).

## Conclusion

In CBA, it is difficult to discover rules involving rare items using a single *minsup* threshold because of the rare item problem. This paper presented the concept of integrating MMSs into established classifiers. Unlike conventional multiple thresholds, the proposed method uses three factors (i.e., *MIS* values for items, *MCS* values for classes, and *MRS* values for rule-items) to determine classification rules.

Experimental results involving six real-world datasets demonstrate that MMSCBA with a maximum likelihood classifier achieves higher accuracy than traditional CBA, especially when the dataset contains a rare item. In addition, the MMSCBA method can resolve the inadequacy of class imbalance and the rare item problem.

Two related issues are worthy of future research. The first is the applicability of this approach to other types of datasets. Previous studies have proposed varied factors that are useful in specific cases; however, these factors are often impractical for analyzing new (or unknown types of) data. The second issue concerns efficiency. Instead of using the Apriori-like algorithm, the proposed method should be extended to other efficient pattern discovery approaches, such as the FP-growth and distributed computing algorithms.

## References

[CR1] Agrawal R, Imieliński T, Swami A (1993). Mining association rules between sets of items in large databases. ACM SIGMOD Rec.

[CR2] Burman P (1989). A comparative study of ordinary cross-validation, v-fold cross-validation and the repeated learning-testing methods. Biometrika.

[CR3] Chen CH, Hong TP, Tseng VS (2009). An improved approach to find membership functions and multiple minimum supports in fuzzy data mining. Expert Syst Appl.

[CR4] Cohen WW (1995) Fast effective rule induction. In: Proceedings of the twelfth international conference on machine learning, pp 115–123

[CR5] Deen AA, Nofal M, Bani-Ahmad S (2010). Classification based on association-rule mining techniques: a general survey and empirical comparative evaluation. Ubiquitous Comput Commun J.

[CR6] Deng H, Runger G, Tuv E, Bannister W (2014). CBC: an associative classifier with a small number of rules. Decis Support Syst.

[CR7] Fernandez-Delgado M, Cernadas E, Barro S, Amorim D (2014). Do we need hundreds of classifiers to solve real world classification problems?. J Mach Learn Res.

[CR8] Guo X, Yin Y, Dong C, Yang G, Zhou G (2008) On the class imbalance problem. In: Proceedings of the fourth international conference on natural computation, pp 192–201

[CR9] Hu YH, Chen YL (2006). Mining association rules with multiple minimum supports: a new mining algorithm and a support tuning mechanism. Decis Support Syst.

[CR10] Hu YH, Chen YL, Lin EH (2007) Classification of time-sequential attributes by using sequential pattern rules. In: Proceedings of the fourth international conference on fuzzy systems and knowledge discovery, pp 735–739

[CR11] Hu YH, Huang TCK, Yang HR, Chen YL (2009). On mining multi-time-interval sequential patterns. Data Knowl Eng.

[CR12] Jain AK, Murty MN, Flynn PJ (1999). Data clustering: a review. ACM Comput Surv.

[CR13] Janssens D, Wets G, Brijs T, Vanhoof K (2005). Adapting the CBA algorithm by means of intensity of implication. Inf Sci.

[CR14] Jyoti S, Ujma A, Dipesh S, Sunita S (2011). Predictive data mining for medical diagnosis: an overview of heart disease prediction. Int J Comput Appl.

[CR15] Kiran RU, Reddy PK (2010) Improved approaches to mine rare association rules in transactional databases. In: Proceedings of the fourth SIGMOD Ph.D. workshop on innovative database research, pp 19–24

[CR16] Lee YC, Hong TP, Lin WY (2005). Mining association rules with multiple minimum supports using maximum constraints. Int J Approx Reason.

[CR17] Li W, Han J, Pei J (2001) CMAR: accurate and efficient classification based on multiple class-association rules. In: Proceedings of IEEE international conference on data mining, pp 369–376

[CR18] Liu B, Ma Y, Wong C (2000). Improving an association rule based classifier. Lect Notes Comput Sci.

[CR19] Liu B, Hsu W, Ma Y (1998) Integrating classification and association rule mining. In: Proceedings of the fourth ACM SIGKDD international conference on knowledge discovery and data mining, pp 80–86

[CR20] Liu B, Hsu W, Ma Y (1999) Mining association rules with multiple minimum supports. In: Proceedings of the fifth ACM SIGKDD international conference on knowledge discovery and data mining, pp 337–341

[CR21] Ngai EWT, Xiu L, Chau D (2009). Application of data mining techniques in customer relationship management: a literature review and classification. Expert Syst Appl.

[CR22] Quinlan JR (1986). Induction of decision trees. Mach Learn.

[CR23] Quinlan JR (1993). C4.5: programs for machine learning.

[CR24] Roddick JF, Spiliopoulou M (2002). A survey of temporal knowledge discovery paradigms and methods. IEEE Trans Knowl Data Eng.

[CR25] Swami DK, Jain RC (2005). A survey of associative classification algorithms. ADIT J Eng.

[CR26] Thabtah FA (2006). Pruning techniques in associative classification: survey and comparison. J Digit Inf Manag.

[CR27] Thabtah FA (2007). A review of associative classification mining. Knowl Eng Rev.

[CR28] Thabtah FA, Cowling P, Peng Y (2004) MMAC: a new multi-class, multi-label associative classification approach. In: Proceedings of the fourth IEEE international conference on data mining, pp 217–224

[CR29] Thabtah FA, Cowling P, Peng Y (2005) MCAR: multi-class classification based on association rule. In: Proceedings of the 3rd ACS/IEEE international conference on computer systems and applications, pp 127–133

[CR30] Vapnik VN (1999). An overview of statistical learning theory. IEEE Trans Neural Netw.

[CR31] Venkatesh E, Thangaraj P (2008). Self-organizing map and multi-layer perceptron neural network based data mining to envisage agriculture cultivation. J Comput Sci.

[CR32] Witten IH, Frank E, Hall MA (2011). Data mining: practical machine learning tools and techniques.

[CR33] Yin X, Han J (2003) CPAR: classification based on predictive association rules. In: Proceedings the third SIAM international conference on data mining, pp 331–335

[CR34] Yoon Y, Lee GG (2013). Two scalable algorithms for associative text classification. Inf Process Manag.

